# Supplementary material for: Protein Enrichment of Donor Breast Milk and Impact on Growth in Very Low Birth Weight Infants
Source: Nutrients. 2021 Aug 20;13(8):2869. doi: 10.3390/nu13082869 (PMC8401419; doi:10.3390/nu13082869)
Supplement: Supplementary file 1 [file nutrients-13-02869-s001.zip › nutrients-1323456-supplementary.pdf]

Supplemental Table S1 – Clinical team rationale for excluding or discontinuing DBM+

Reason for Exclusion	Number of Infants
Excluded from DBM+ from admission	
Critical Illness	10
Gestational Age	
22 weeks	3
23 weeks	4
24 weeks	2
Initially eligible for DBM+, then discontinued	
Acute Kidney Injury	2
Electrolyte Abnormalities	1
Abdominal Exam	2*
Patent Ductus Arteriosus	1
Other Clinical Illness	2

* 1 infant received only MBM in the 10 days prior to change in exam.

DBM+ – protein-enriched donor breast milk
MBM – maternal breast milk
